# Swin transformer-based GAN for multi-modal medical image translation

**DOI:** 10.3389/fonc.2022.942511

**Published:** 2022-08-08

**Authors:** Shouang Yan, Chengyan Wang, Weibo Chen, Jun Lyu

**Affiliations:** ^1^ School of Computer and Control Engineering, Yantai University, Yantai, China; ^2^ Human Phenome Institute, Fudan University, Shanghai, China; ^3^ Philips Healthcare, Shanghai, China

**Keywords:** magnetic resonance imaging, Swin Transformer, generative adversarial network, multi-modal, medical image translation frontiers

## Abstract

Medical image-to-image translation is considered a new direction with many potential applications in the medical field. The medical image-to-image translation is dominated by two models, including supervised Pix2Pix and unsupervised cyclic-consistency generative adversarial network (GAN). However, existing methods still have two shortcomings: 1) the Pix2Pix requires paired and pixel-aligned images, which are difficult to acquire. Nevertheless, the optimum output of the cycle-consistency model may not be unique. 2) They are still deficient in capturing the global features and modeling long-distance interactions, which are critical for regions with complex anatomical structures. We propose a Swin Transformer-based GAN for Multi-Modal Medical Image Translation, named MMTrans. Specifically, MMTrans consists of a generator, a registration network, and a discriminator. The Swin Transformer-based generator enables to generate images with the same content as source modality images and similar style information of target modality images. The encoder part of the registration network, based on Swin Transformer, is utilized to predict deformable vector fields. The convolution-based discriminator determines whether the target modality images are similar to the generator or from the real images. Extensive experiments conducted using the public dataset and clinical datasets showed that our network outperformed other advanced medical image translation methods in both aligned and unpaired datasets and has great potential to be applied in clinical applications.

## 1 Introduction

Magnetic resonance imaging (MRI) has become one of the most widely used and powerful tools for clinical diagnosis and treatment nowadays. Since it is a non-invasive imaging method, MRI can yield multiple tissue contrasts by applying various pulse sequences and parameters without exposing the subject to radiation, thus generating multi-modal MR images of the same anatomical structure (1, 2). Some common modalities are T1-weighted (T1), T2-weighted (T2), T1 with contrast enhancement (T1c), and T2 fluid-attenuated inversion recovery (FLAIR) (3). Each modality has its own specific pathological features. The complementary information about tissue morphology allows physicians to diagnose with greater accuracy and confidence. However, many factors, such as limited scanning time and the expensive cost, hinder multi-modal MR imaging. Therefore, there has been growing interest in retrospectively synthesizing missing or corrupted modalities from other successfully acquired ones. Bypassing the cost of additional scanning, this kind of medical image-to-image translation method not only facilitates the reliability of clinical diagnosis but also promotes follow-up image analysis tasks such as registration (4, 5) and segmentation (6, 7).

Recently, various deep learning methods have been exploited to solve the problem of medical image-to-image translation in an end-to-end manner. Previous studies (8) have demonstrated that generative adversarial network (GAN) has significant potential in solving image-to-image translation problems. GAN is a framework that simultaneously trains a generator G and a discriminator D by an adversarial process. During the training process, the generator is used to translate the distribution of source modality MRIs to the distribution of target modality MRIs. The discriminator is used to identify whether target modality MRIs are likely from the generator or the real data. These GAN-based approaches can be broadly divided into two categories. One refers to the supervised Pix2Pix (8–12) GAN approach, which utilizes paired images from the source and target modalities. However, it relies on paired and pixel-aligned images, which may not always be possible due to respiratory movements or anatomical changes between the times when multi-modality images are scanned. For instance, Isola et al. proposed (13) a conditional adversarial network for image-to-image translation tasks. A three-dimensional (3D) auto-context-based locality adaptive multi-modality GAN model (LA-GANs) (9) is developed to synthesize the high-quality FDG PET image from the low-dose one with the help of MRIs. Zhan et al. (10) utilized a conditional GAN for multimodal MRI synthesis by modeling the non-linear mapping between input and output. The other category involves unsupervised cycle-consistency GAN (14–16), which can be used for misaligned images through a cycle-consistency loss. However, it is known that the cycle-consistency framework may have multiple solutions (17, 18), indicating that the results may not be accurate and sensitive to perturbation. To solve the mentioned problems, Kong et al. (19) proposed RegGAN, which incorporates a registration network and regards the misaligned target images as noisy labels.

However, the convolution kernel usually has a limited receptive field and thus cannot capture long-range dependencies, which are essential for MR image-to-image translation. Nowadays, vision transformer (20) is capable of modeling global interactions between contexts and has promising performance in MRI restoration (21, 22), segmentation (23, 24), and registration (25, 26). Nevertheless, vision transformers for image restoration need to divide the input image into small patches of fixed size, which may introduce border artifacts around each small patch in the restored images. To solve this problem, Swin Transformer (27) has been proposed to solve many vision problems (28, 29) since it integrates the advantages of both the convolutional neural network (CNN) and the self-attention mechanism (30) with shifted windows.

**Figure 1:**
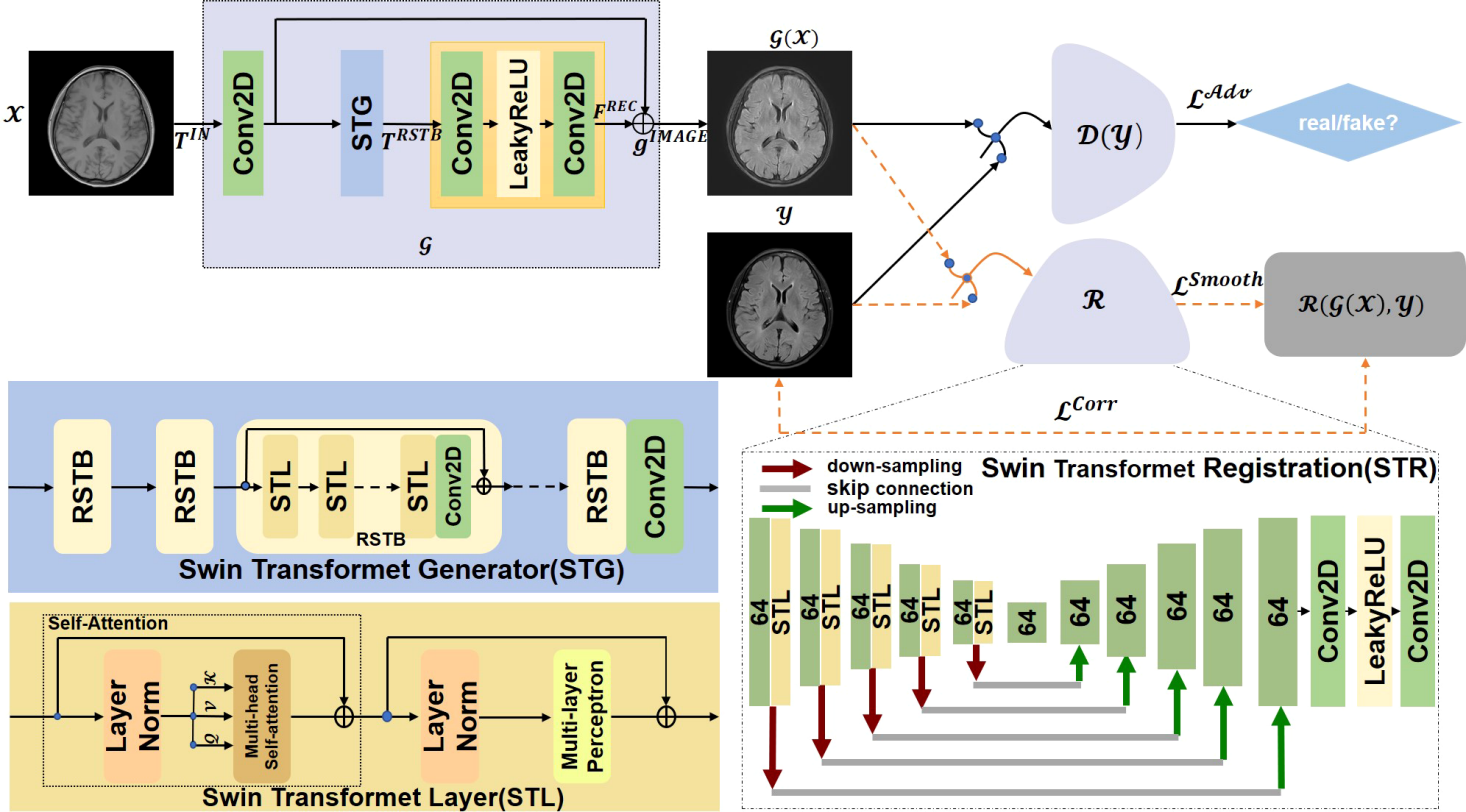
The overall architecture of the proposed MMTrans, including the Swin Transformer Generator (STG) for target image translation and the Swin Transformer Registration (STR) for mismatch correction.

In this paper, we propose a Swin Transformer-based GAN for Multi-Modal Medical Image Translation, called MMTrans. More specifically, our framework consists of three modules: a Generator, a Registration Network, and a Discriminator. The Generator is based on the framework of SwinIR (30), which is utilized to generate images with the same content as source modality images and the similar style information of target modality images. The registration network is a Swin Transformer model, which is trained to predict the deformable vector field (DVF). For paired images, we assume that there exists a tiny mismatch between the source domain images and the target domain images. Therefore, the mismatch can be corrected by the registration network. For unpaired images, as shown in **Figure 1**, the *G*( *x* ) generates images with the same morphology as T1 and the same style as T2, while *ℛ*( *G*( *x* ),*y* ) represents the image with the same style and the same morphology of T2. The discriminator, a CNN model, determines whether the target modality images are similar to the generator or from the real images. Extensive experiments on paired and unpaired public and clinical data demonstrated that the proposed MMTrans outperforms state-of-the-art approaches and has great potential to be applied in clinical practice.

This paper’s sections were arranged as follows: in Section 2, we elaborate on the proposed MMTrans framework, including Swin Transformer Generator, Swin Transformer Registration, Swin Transformer Layer, and the loss function. In Section 3, we give the details of the experiment. Then we present and discuss the experimental results in Section 4 and finally summarize the conclusions in Section 5.

## 2 Methods

The task of this study is to synthesize translated modalities from given modalities in MR images. In order to obtain better performance, we propose a Swin Transformer-based GAN for multi-modal MRI translation. **Figure 1** shows the flowchart of the whole framework. In this section, we will introduce in detail the Swin Transformer Generator, Swin Transformer Registration, Swin Transformer Layer, and loss functions.

### 2.1 Swin Transformer layer

The most significant improvement and development of the Swin Transformer to the transformer are replacing the previous standard multiple self-attention (MSA) module with a shift window-based module, with no substantial changes to the other layers. Each Swin Transformer block consisted of layer norm, multi-headed self-focused modules, residual connections, and a two-level MLP with GELU non-linearity. Similar to previous reports (31, 32), self-concern was calculated as follows:


(1) $$Attention\left(Q,K,V\right)=SoftMax\left(\frac{Q\;K{\;}^{T}}{\sqrt{d}}+ℬ\right)V$$

We represented the query, key, and value as *Q*, *K*, *and* *V ∈ R* ^*m* ^2×*d*^^ respectively; *m* ^2^ represents the number of patches in the window, while *ℬ* depicts the dimension of the query or key. The values in *ℬ* were selected from $\hat{ℬ}\in R^{\left(2m-1\right)\times \left(2m+1\right)}$ , the bias matrix.

### 2.2 Swin Transformer generator

Currently, the models that can only be applied to specific scenes or from minimal modeling capabilities generally perform well in image translation. Most direct training CNNs first encode the image as a high-level feature representation and then decode it to full spatial resolution. Thus, these models are challenging to apply in medical imaging. Image-to-image translation is ultimately about inputting a high-dimensional input tensor and then corresponding this tensor to an output tensor with a different appearance but identical in basic structure. In the image-to-image conversion, shallow and deep characteristics are extracted from the input and the actual images to achieve high-quality image translation. To achieve this goal, a Swin Transformer-based generative network for target image generation was constructed. By using the transformer to introduce a self-attention mechanism into the encoder design, deep hierarchical representations were extracted with rich remote dependencies from both the target and reference images, which performed the translation task more efficiently and accurately. As shown in **Figure 1**, Swin Transformer Generator (STG) consisted of multiple residual Swin Transformer blocks (RSTBs), each using various Swin Transformer Layers (STLs) for local attention and cross-window interaction learning, and the RSTB used residual learning to ensure the stability of feature extraction and 3 × 3 convolutional layers between RSTBs and STLs for feature enhancement. The feature extraction process of RSTBs was expressed as follows:


(2) $$T^{RSTB}=Conv\left(F^{STL}\right)+T^{IN}$$

where *F^STL^* denotes the model generated from STLs; Conv means 3 × 3 Conv2D, and *T^IN^* represents the input feature of RSTBs. As shown in **Figure 1**, each STL consisted of multi-headed self-attentive blocks and multi-layer perception. In this study, the number of RSTBs and STLs in STG is set at four and six, respectively. As shown in **Figure 1**, the STG consisted of multiple RSTBs, each using various STLs for local attention and cross-window interaction learning, and each RSTB used residual learning to ensure the stability of feature extraction and 3 × 3 convolutional layers between RSTB and STL for feature enhancement. The generation section was defined as follows:


(3) $$G^{IMAGE}=F^{REC}\left(T^{RSTB}\right)$$

where *F^REC^* represents the function of the recovery module through the long-skit connection. We used the STG module to feed the low-frequency information wholly and directly into the recovery module to extract high-frequency data from the depth features. In the recovery, the subpixel convolutional layer was adopted by us.

### 2.3 Swin Transformer registration

Compared with the traditional image translation tasks, image translation in the medical field is more difficult because of the large amount of detailed medical information contained in the structure of the medical images. This information is inevitably lost during training. The approach in the current work required the construction of a network specifically for the specific medical image translation task to solve this problem. A primary registration network was added to the image translation work in this study of RegGAN (19). Therefore, it was feasible to use the registration networks to train generators in the medical image translation process. We referred to a previous study (26) using a U-shaped structure as the structure of the registration network, both through the encoder-decoder paradigm, to achieve a smooth and gradual transition from the image to the registration. Unlike the previous study (33) and its variants, the encoder part of our Swin Transformer Registration (STR) architecture better learned the display’s global and remote semantic information interaction. In our STR network, a Swin Transformer Layer was added to the encoder part to perform the feature extraction process, which improved the performance of our network by obtaining better global information. We also used alternative up-sampling, general convolution, and jump junction, which allowed the image features extracted in the encoder part of the network to be passed directly to the decoder section. We adopted a standard convolutional layer with an available kernel size of 3 × 3 and a stride size of 2 × 2 for this work. We added a LeakyRelu layer with the parameters equal to 0.2 behind the standard convolutional layer. As shown in the STR section of **Figure 1**, each rectangle represents a two-dimensional image to better train the registration architecture SWR for target image generation. The numbers of rectangles represented how many filter convolutions were used in the process. The down-sampling operation was represented with bright red arrows, and the up-sampling procedure was represented with green arrows; the gray connecting line represented the jump connection between the encoder and the decoder. Finally, the full-resolution image was further refined after two layers of standard convolution. Our results showed that this registration network could perform well in the task of image translation.

### 2.4. Loss functions

First, the underlying network framework involves GANs (34), where the generator *G* and the discriminator *D* are continuously trained to play against each other during the training process and are eventually introduced to the desired ideal state. In this process, we trained the generator to produce the medical target image *G*( *x* ) ideally from the input *x* image. Quite differently, this was the discriminator in our network, which was continuously trained to separate from the ground truth image *y* or the perfect target medical image *G*( *x* ) developed by the generator. The adversarial loss function was as follows:


(4) $$\begin{array}{c} min\\ G \end{array}\begin{array}{c} max\\ D \end{array}{ℒ}^{Adv}\left(G,D\right)=\epsilon_{y}\left[\text{log}\left(D\left(y\right)\right)\right]+\epsilon_{x}\left[\text{log}\left(1-D\left(G\left(x\right)\right)\right)\right]$$

After experiencing the target medical image *G*( *x* ) produced after generating the adversarial network, we added the registration network *ℛ* as a label noise model to correct the generated target image *G*( *x* ) to achieve better translation. The correction loss is shown in Equation 5:


(5) $$\begin{array}{c} min\\ G,ℛ \end{array}{ℒ}^{Corr}\left(G,ℛ\right)=\epsilon_{x,y}\left[{∥y-G\left(x\right)\circ ℛ\left(G\left(x\right),y\right)∥}_{1}\right]$$

In Equation 5, we used *ℛ*( *G*( *x* ),*y* ) to represent the deformation field operation and we used ° to describe the resampling operation. Our network’s registration network was constructed based on the U-Net (35). In Equation 6, the smoothness of the deformation field was evaluated by the loss function, and the gradient of the deformation field was minimized.


(6) $$\begin{array}{c} min\\ ℛ \end{array}{ℒ}^{Smooth}\left(ℛ\right)=\epsilon_{x,y}\left[{∥\nabla ℛ\left(G\left(x\right),y\right)∥}^{2}\right]$$

Finally, the total loss function of our network is shown in Equation 7, which has three components.


(7) $$\begin{array}{c} min\\ G,ℛ \end{array}\begin{array}{c} max\\ D \end{array}{ℒ}^{Total}\left(G,ℛ,D\right)=\kappa {ℒ}^{Adv}+\lambda {ℒ}^{Corr}+\mu {ℒ}^{smooth}$$

## 3 Experiment

In the following paragraph, we introduced the experimental setup, including the used data and practical methods, evaluation indicators, and some implementation details.

### 3.1 Dataset

We employ three different datasets to evaluate our method, as shown in **Table 1**:

- Open access BraTs2018 (36) dataset. The dataset contains multi-contrast images, such as T1 and T2. BraTs2018 was selected because the original images were paired and well aligned.
- Public fastMRI (37) dataset with paired multi-contrast knee DICOM images. We only used the coronal PD and PD-FS images.
- The clinical brain MRI dataset was acquired with a 3T Philips Ingenia MRI system (Philips Healthcare, Best, the Netherlands) scanner, including T1-weighted (T1W) and FLAIR imaging. The dataset consists of 17 healthy subjects and five patients. All subjects gave their informed consent for inclusion before they participated in the study with approval from the local institutional review board (in accordance with the Declaration of Helsinki). The institutional review board has approved the MRI scanning.

**Table 1:** Three datasets and number of images for training validation and test.

Datasets	BraTs2018	fastMRI	Clinical brain MRI
Original image	T1	PD	T1
Target image	T2	PD-FS	T2
Train/valid/test	1,000/300/300	300/80/80	500/150/150

When training on paired images, all the MRIs were well aligned and normalized into the range of [0, 1]. However, when training on unpaired images, we randomly sample one image from T1 and the other one from T2.

### 3.2 Implementation details

Our proposal was implemented in PyTorch with an NVIDIA Tesla V100 GPU (4 × 16 GB). The optimizer used was Adam at a learning rate of 1e−4 to test all the developed methods. Each training process protected 80 epochs, and the weights of the different loss functions were κ = 1, *λ*= 20, and *μ*= 10. The error maps are calculated by calculating the absolute difference between the generated images with the ground truth images. The error maps are calculated by calculating the absolute difference between the generated images with the ground truth images.

### 3.3 Comparison methods and evaluation metrics

Two board-certified radiologists (with 7 and 10 years of experience) independently reviewed the images synthesized by all the comparison methods. The synthesized images were anonymized, and the order of the image translation methods was randomized. Three types of image quality measures (overall image quality, image contrast, and structure outline) were scored with 5-point Likert scale, 5-point Likert scale, and 3-point scale. The 5-point Likert scale for overall image quality and image contrast was as follows: 1, unacceptable; 2, poor; 3, acceptable; 4, good; and 5, excellent. The 3-point scale for structure outline was as follows: 1, indistinct outline; 2, perceptible outline; and 3, sharp outline. One-tailed Wilcoxon signed-rank tests based on the ratings of two radiologists were used to test the difference between synthesized images of different methods and the ground truth images. The significance level was set as 0.01.

## 4 Experimental results

### 4.1 Hyperparameter selection

**Figure 2:**
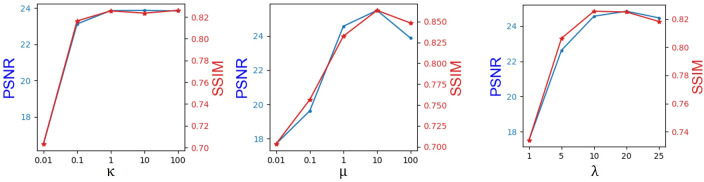
Hyperparameter selection results in the objective function.

We obtain the hyperparameters *κ*, *λ*, and *μ* by the greedy method, as shown in **Figure 2**. Note that the hyperparameter tuning is performed in the BraTs2018 dataset. The coefficient of the adversarial loss was first adjusted. When *κ* increased from 0.01 to 1, PSNR and SSIM show a growth tendency. However, it can be seen that both PSNR and SSIM decrease slightly when *κ* boosts from 1.0 to 100. Thus, *κ* is set to 1. When *κ* is fixed, *μ* is increased from 0.01 to 100. As can be seen, the PSNR and SSIM values keep growing until *μ* reaches 10. When *μ* >10, both PSNR and SSIM values show a declining trend. Therefore, we set *μ* to 10. In practice, we found that it is adequate to set *λ* from 1 to 20 such that the magnitude of different loss terms is balanced into similar scales. As shown in **Figure 2**, we set *λ* to 20. Moreover, it has been demonstrated that adjusting the hyperparameter determination order will not affect the final hyperparameter setting.

### 4.2 Qualitative results

**Figure 3:**
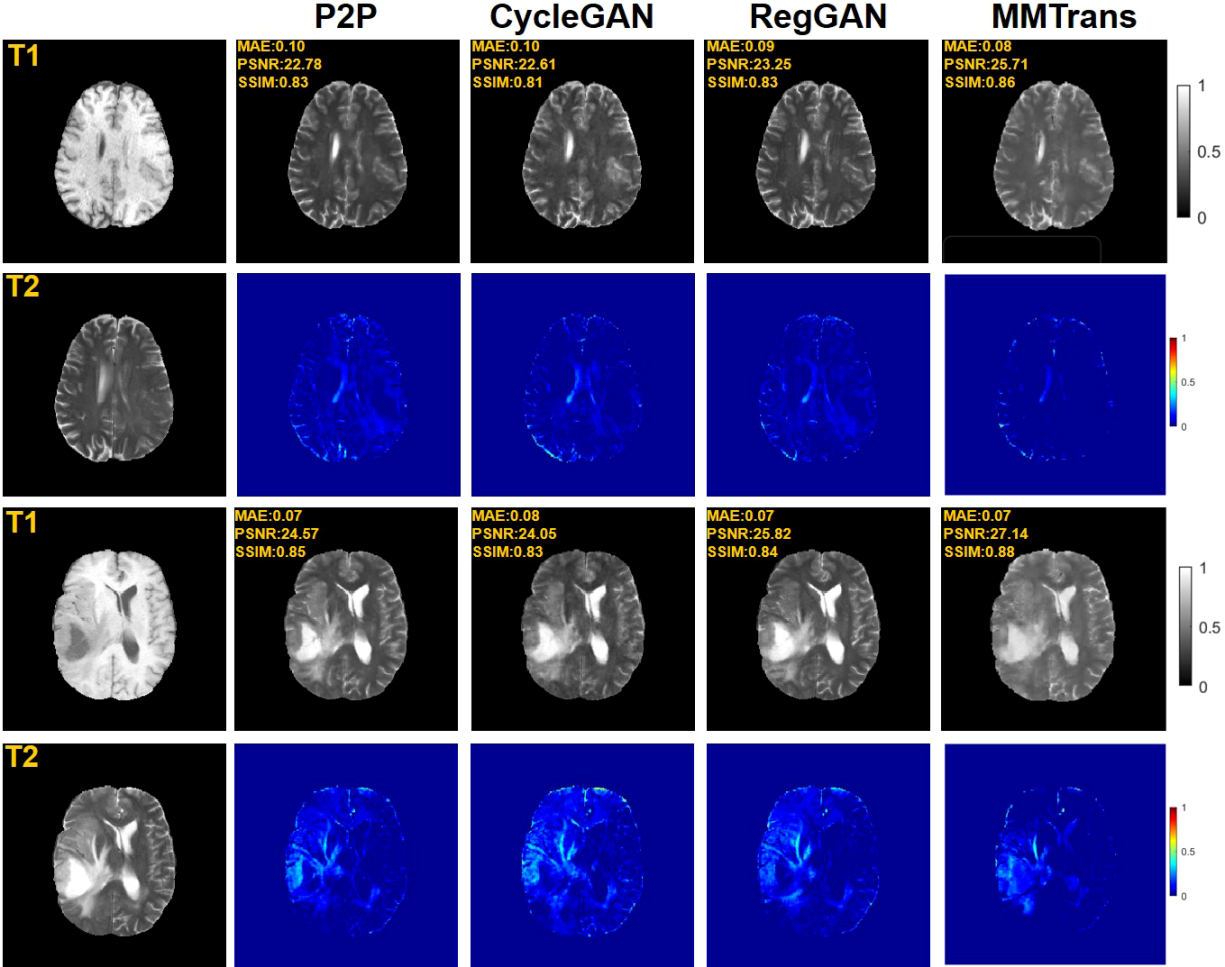
Qualitative results of T1 modality translation to T2 modality using the BraTs2018 paired dataset with different translation methods, displaying translation images and corresponding error map.

**Figure 4:**
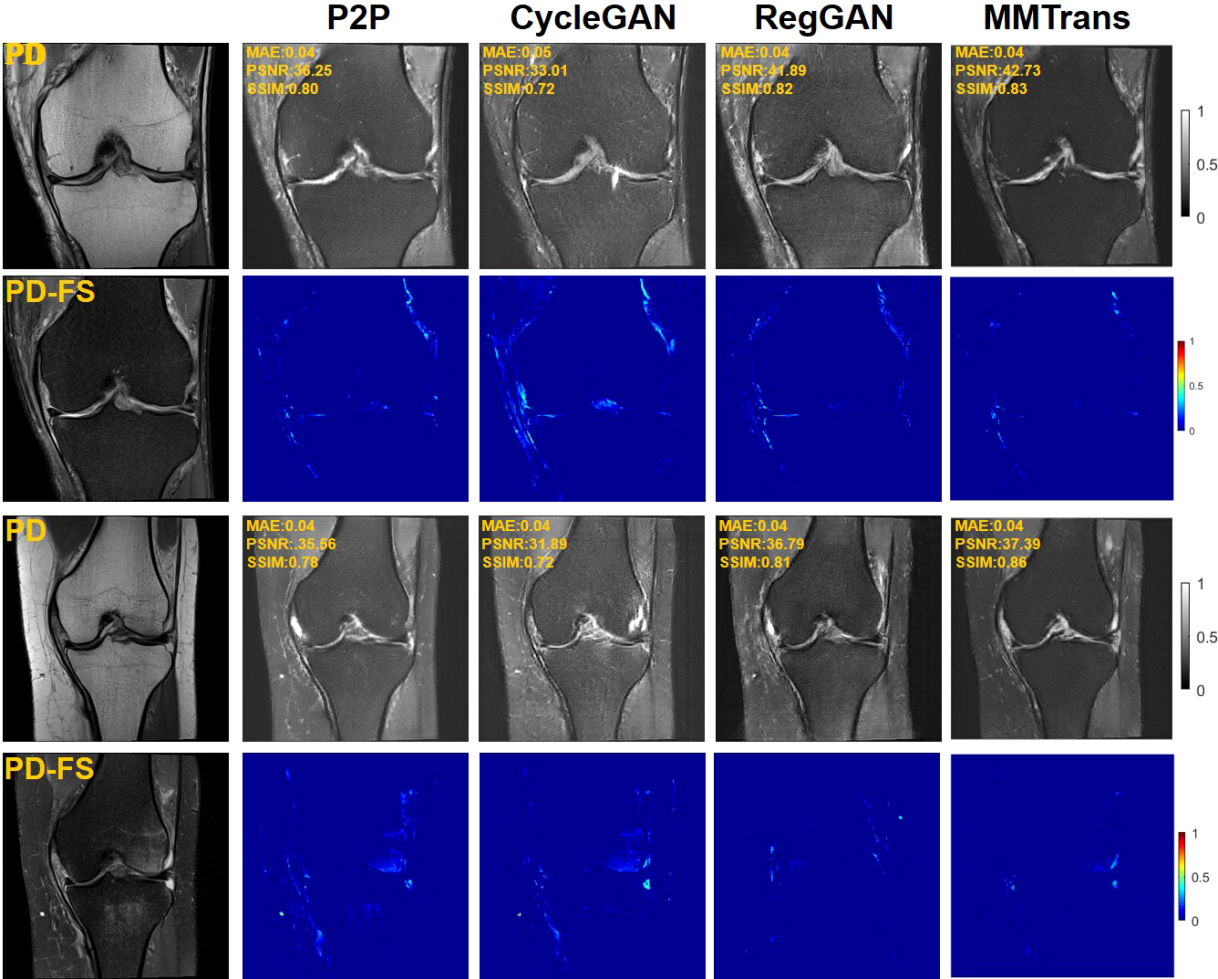
Qualitative results of different translation methods from PD to PD-FS using public fastMRI dataset, displaying translation images and corresponding error map.

**Figure 5:**
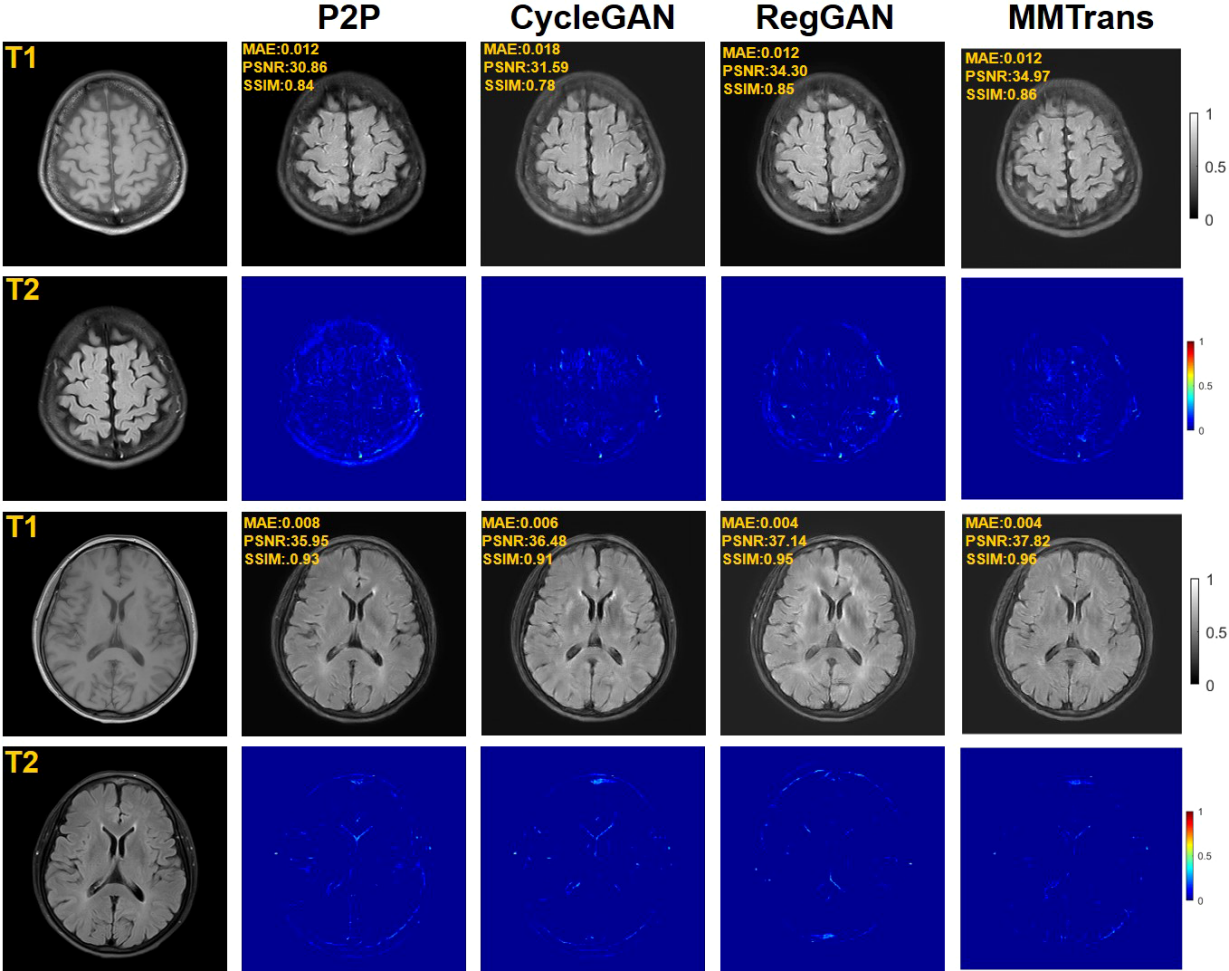
Qualitative results of different translation methods for translating T2 from T1 using the paired clinical brain MRI dataset, showing translation images and corresponding error maps.

**Figure 6:**
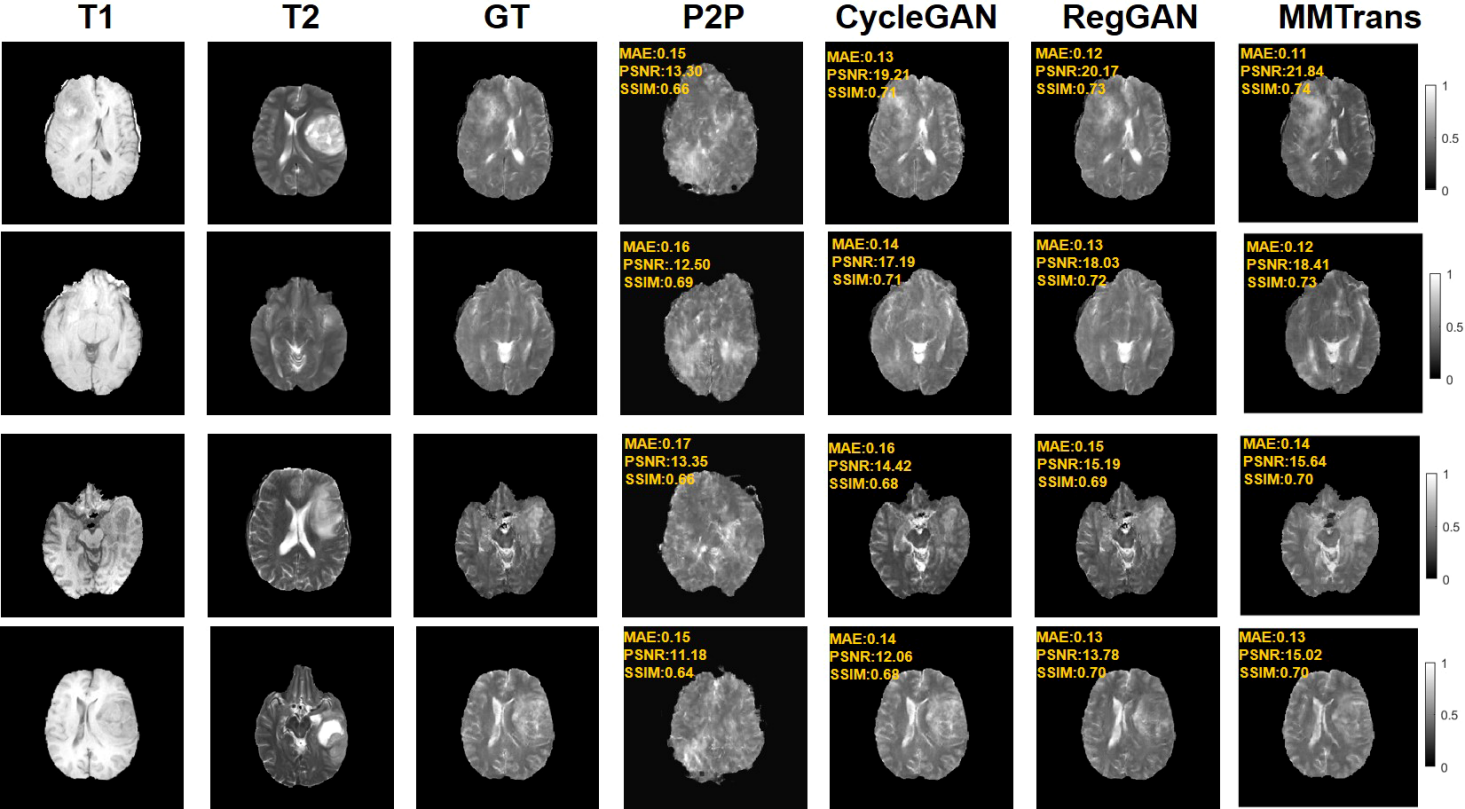
Qualitative results of different translation methods for synthesizing T2 from T1 on unpaired BraTs2018 dataset.

**Figure 7:**
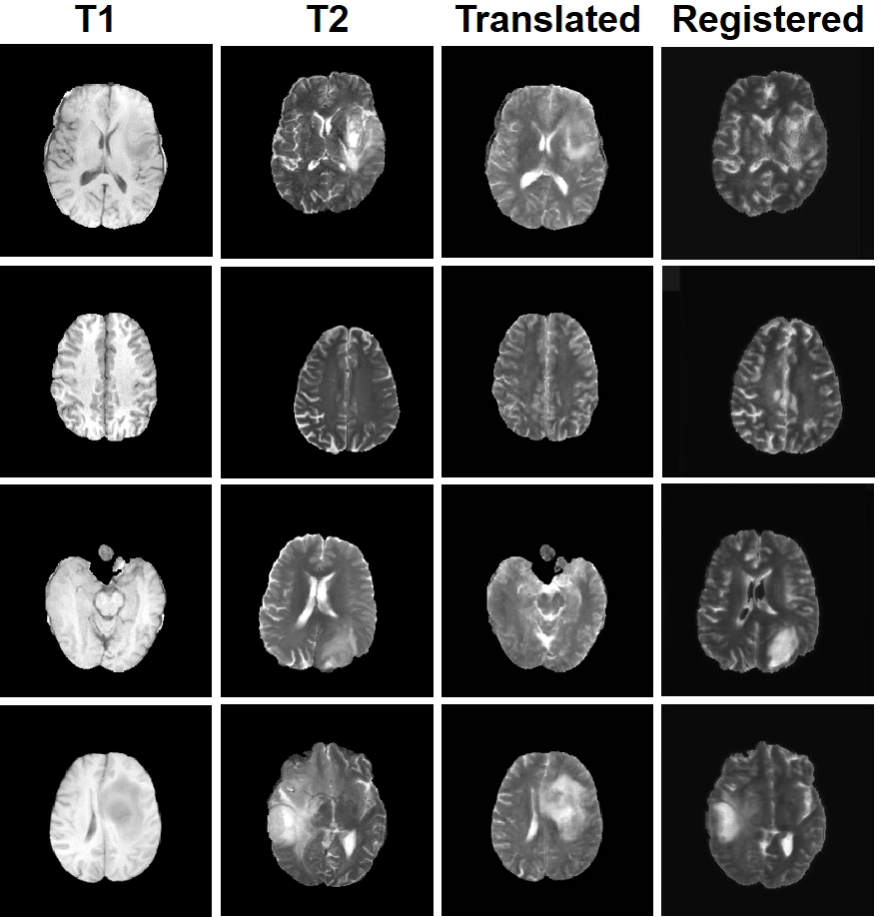
Display of MMTrans output on unpaired data. T1 and T2 are unpaired images. *Translated* represents the translation result of T1 to T2. *Registered* represents the registration result of the translated images.

Four tasks are used to evaluate and test the proposed image translation model. First, on the BraTs2018 paired dataset, the T2 modality of the T1 translation was used. On the public fastMRI dataset, converting PD modality to PD-FS modality is performed as a second task. The third task was to convert T1 mode to T2 mode on a clinical brain MRI paired dataset. Finally, on the BraTs2018 unpaired dataset, the T2 modality of the T1 translation was used. The translation performance of MMTrans is first evaluated on BraTs2018 paired images; **Figure 3** shows the comparison of the translation method proposed in the paired dataset BraTs2018 with other state-of-the-art. Clearly, our proposed method produces better translation results, which are valuable in clinical applications. For the second task (implementing PD image to PD-FS image translation using public fastMRI datasets), **Figure 4** shows that our model generates target images with higher quality and better clarity as compared to other models. Admittedly, in the qualitative comparison results shown in **Figure 5**, the best performance of our method is also achieved in the third task (conversion from T1 mode to T2 mode images on top of the clinical brain MRI dataset). Lastly, the performance was evaluated using the BraTs2018 unpaired dataset; the results in **Figure 6** show that our proposed MMTrans achieves the best translation performance. In **Figure 7**, we show how MMTrans corrects unpaired data. It can be seen that MMTrans will try its best to eliminate the influence of unpaired through registration.

### 4.3 Quantitative results

**Figure 8:**
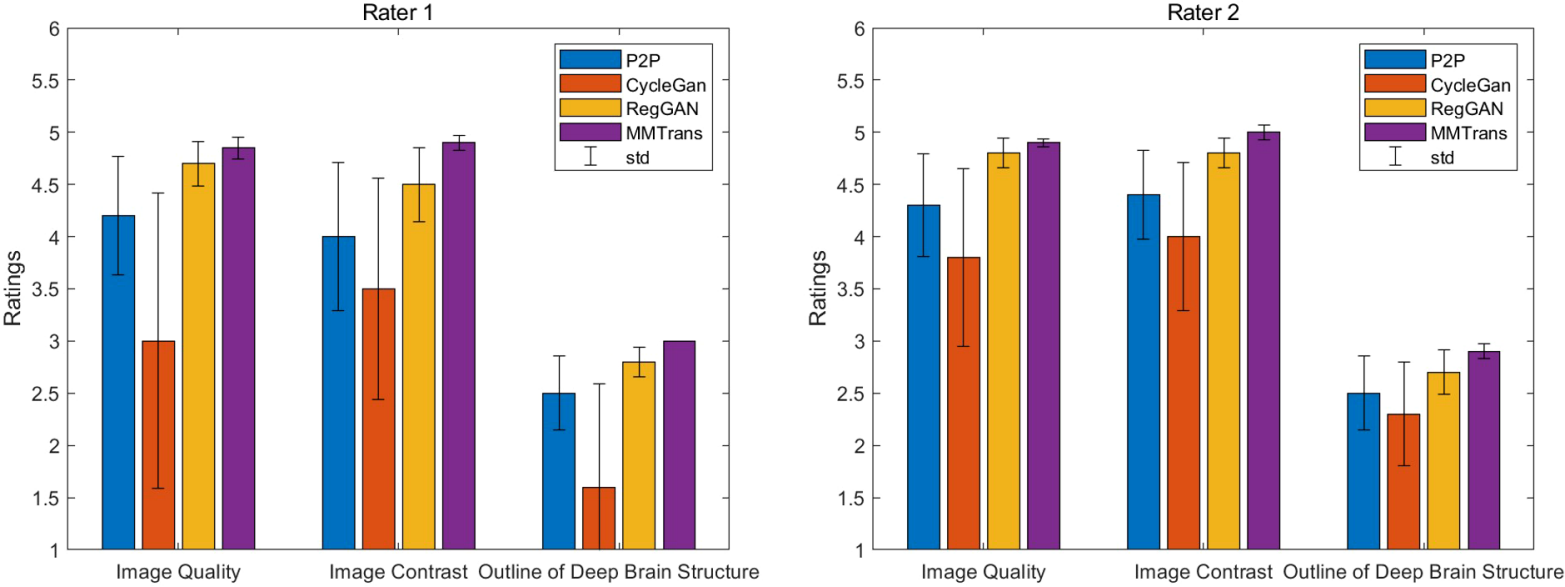
Two board-certified radiologists (7 and 10 years of experience) independently reviewed the results of images synthesized by all contrast methods.

The values of quantitative metrics for the two raters are shown in **Table 2** and **Figure 8**. Both raters agreed that our translated images significantly improved overall quality (*p* < 0.01), image contrast (*p* < 0.01), and deep brain structure contours (*p* < 0.01). Meanwhile, our synthetic T2 images and real T2 images were not significantly different for all measures of image quality (all *p* > 0.01)..

**Table 2:** Compare the mean scores of translated images given by P2P images, CycleGAN images, RegGAN images, and MMTrans images.

	Ratings (mean ± standard deviation)				*p*-Value MMTrans
	P2P	CycleGAN	RegGAN	MMTrans	GT
Quality	4.25 ± 0.53	3.40 ± 1.13	4.75 ± 0.17	4.88 ± 0.07	0.029
Contrast	4.20 ± 0.56	3.75 ± 0.88	4.65 ± 0.24	4.95 ± 0.07	0.015
Outline	2.50 ± 0.35	1.95 ± 0.74	2.75 ± 0.17	2.95 ± 0.03	0.023

The translation performance of MMTrans is first evaluated on BraTs2018 paired images. **Table 3** shows the results of quantitative evaluations on the four tasks. The first is evaluated on BraTs2018 paired images; **Table 3** shows that our model dominates the PSNR, NAME, and SSIM metrics, indicating that our model achieves better target image translation. Based on **Table 3**, we can find that our model method outperforms other image translation methods on the fastMRI public dataset, especially in image contrast restoration. Admittedly, quantitative results on the third task (translation from T1 modality to T2 modality images on a clinical brain MRI paired dataset) suggest that our approach is the best solution. Finally, the BraTs2018 unaligned dataset is used to evaluate the performance of using T1 image transfer to T2 modality; the quantitative evaluation results of this task are shown in **Table 3**. Comparing the three evaluation metrics in **Table 3**, MMTrans performs better.

**Table 3:** Quantitative metrics results (mean and standard deviation) on different datasets in terms of PSNR, MAE, and SSIM.

Dataset	BraTs2018 (paired)			fastMRI (paired)		
Metrics	PSNR	MAE (10^-2^)	SSIM (10^-2^)	PSNR	MAE (10^-2^)	SSIM (10^-2^)
P2P	23.80 ± 3.81*	8.27 ± 1.90*	81.67 ± 3.80*	35.36 ± 2.37*	4.31 ± 0.90*	76.17 ± 6.40*
CycleGAN	22.59 ± 3.26*	8.85 ± 1.90*	80.64 ± 3.40*	34.20 ± 2.64*	4.38 ± 1.30*	74.27 ± 8.60*
RegGAN	24.08 ± 3.38*	8.18 ± 1.90*	82.83 ± 3.60*	37.28 ± 2.05*	4.28 ± 1.10*	80.18 ± 6.40*
MMTrans	**24.83 ± 3.36**	**8.06 ± 1.80**	**83.95 ± 3.70**	**39.37 ± 2.38**	**4.10 ± 1.20**	**81.17 ± 7.00**
Dataset	Clinical brain MRI (paired)			BraTs2018 (unpaired)		
P2P	34.67 ± 4.08*	1.62 ± 1.10*	84.86 ± 8.00*	12.57 ± 2.8*	19.36 ± 3.50*	67.90 ± 1.90*
CycleGAN	34.23 ± 2.74*	1.67 ± 0.10*	83.92 ± 7.30*	13.34 ± 2.11*	18.11 ± 3.40*	70.25 ± 2.50*
RegGAN	36.13 ± 3.69*	1.34 ± 0.80*	86.37 ± 8.00*	14.02 ± 2.11*	17.82 ± 2.80*	71.17 ± 2.40*
MMTrans	**36.70 ± 3.13**	**1.28 ± 0.60**	**87.49 ± 8.00**	**14.86 ± 2.34**	**16.26 ± 2.80**	**73.09 ± 2.90**

The best quantitative metrics results are marked in bold.

*Significant difference between different comparison methods and our proposed MMTrans.

### 4.4 Ablation study

**Figure 9:**
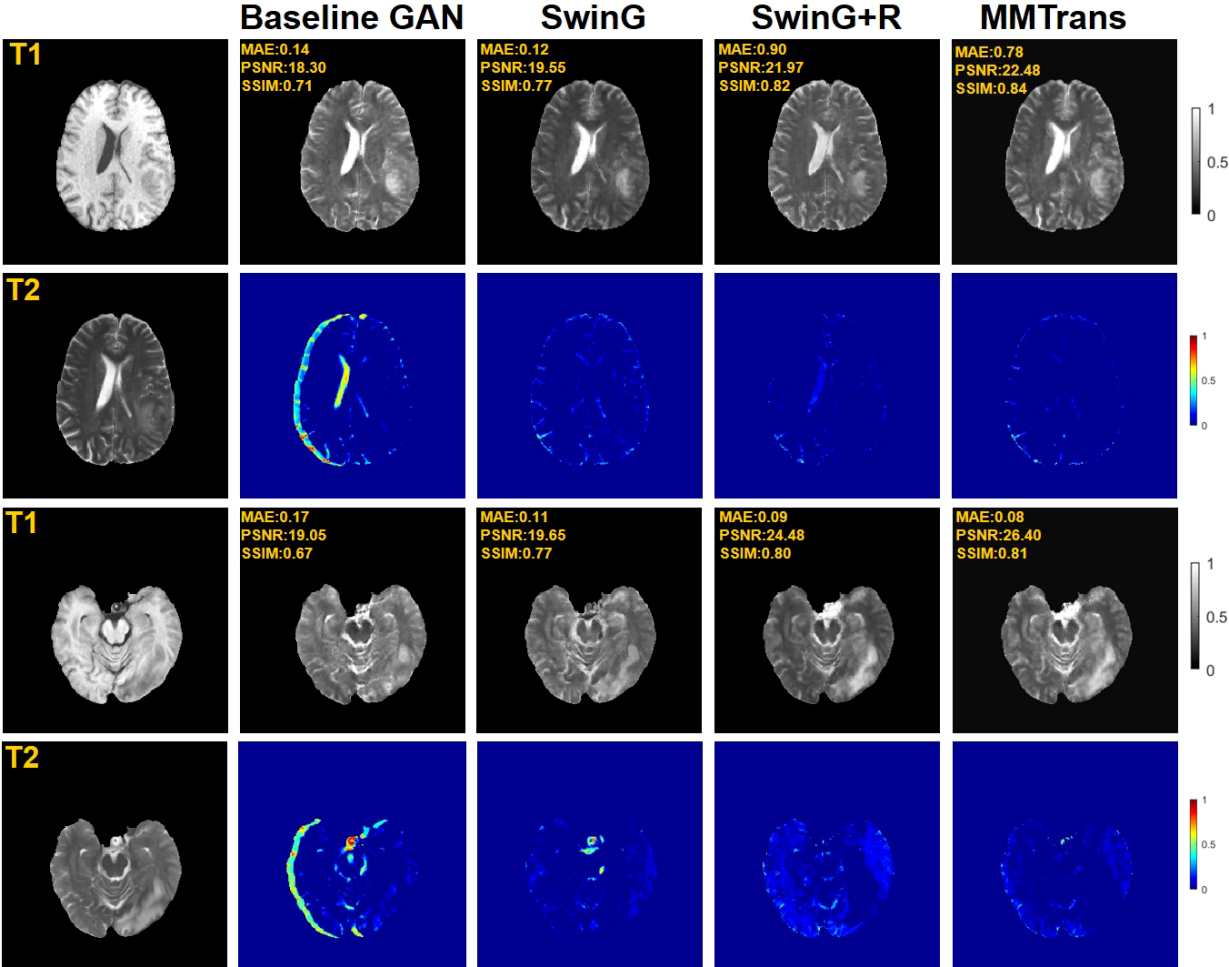
Qualitative results in ablation studies under different scenarios on the BraTs2018 dataset.

**Figure 10:**
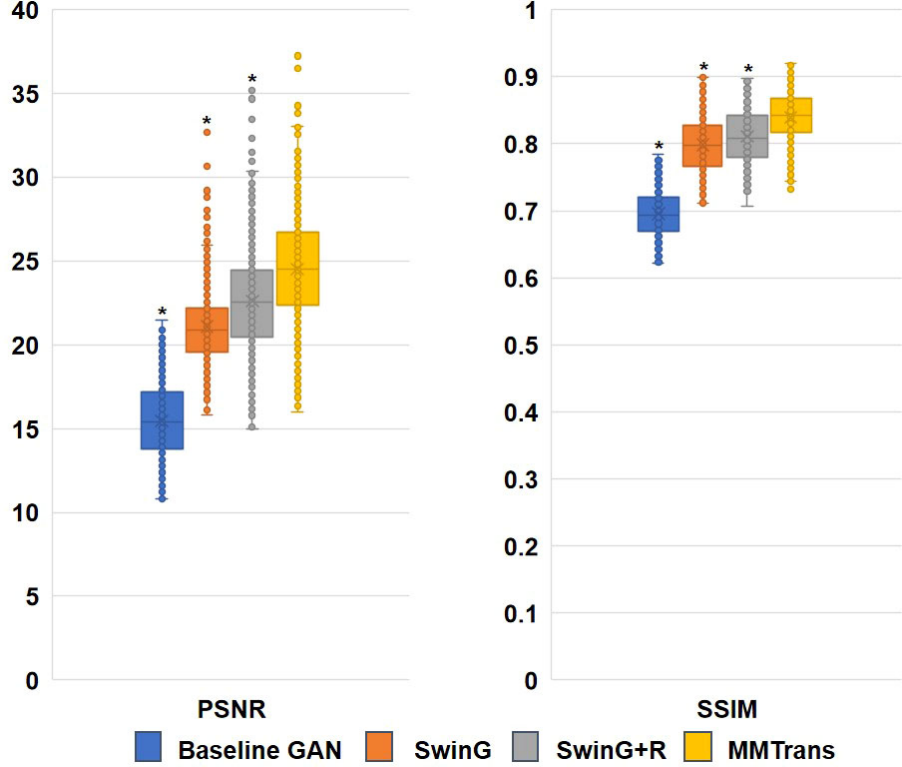
Ablation study results with different scenarios on the BraTs2018 dataset. *Significant difference between different comparison methods and our proposed MMTrans (*p* < 0.01).

**Figure 11:**
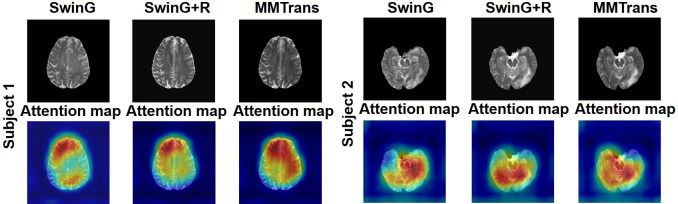
The learned attention map. Visualization of attention maps in SwinG, SwinG+R, and MMTrans. Blue and red values represent low and high response values, respectively.

To analyze the impact of STL modules in our proposed architecture, we perform an ablation study for four different scenarios: 1) baseline GAN: both G and R consist of the convolutional layer. 2) SwinG: we disable the registration module and only add the STL module to the G network. 3) SwinG+R: the STL module is added to generator G, while the registration R is without the STL module. 4) MMTrans (ours): both G and R adopt STL modules. The qualitative and quantitative results are shown in **Figures 9**, **10**, respectively. First, adding the registration network (+R) obviously improves the performance of the method. Second, the residual Swin Transformer group enables better modeling of long-range dependency of MRIs since MRIs often have repeating visual patterns and similar structures. As shown in **Figure 11**, adding the registration R network makes the translation more accurate, and the STL modules are added to generator G and registration R, which can learn more features of MRIs. Therefore, the proposed MMTrans can be regarded as a better translation scheme.

## 5 Discussion

In contrast to previous image-to-image translation methods, the Pix2Pix must be trained with enough paired data to generate a clear image of the pathological target. However, unavoidable physical factors during the MR image acquisition, including respiratory motion or anatomical variations between the acquired pairs of photos, make it very difficult to achieve precisely matched MR data from the same individual. Even with the excellent performance of the Pix2Pix mode, it must require a large number of pixels to align medical images, which is a very time-consuming task for MRI. Specific to cyclic consistency, it is impossible to meet the medical image requirements at the accuracy level. Applying image translation to medical imaging requires a change in style between two images and, more importantly, the ability to achieve higher-resolution conversions between specific pairs of medical images. The result should be unique, and the translated image must maximize the anatomical features of the original image. Our model performed best with paired and unpaired data combining image translation with the Swin Transformer.

This work proposed a framework that can translate the medical image patterns accurately. In medical image translation, convolutional operations have a fixed localization, making it difficult for CNN-based approaches to learn the display’s global and remote semantic information interaction. In other words, because the convolutional kernel can be considered as a small patch in which the acquired features are of local information, the global data are lost when remote dependency modeling training is performed at its location, which also leads to the inability to obtain the anatomical details contained inside the image during the translation process. With the help of the Swin Transformer, the generator part of our network was built based on the work, where the input medical image is segmented into non-overlapping image patches; each patch can be referred to as a token, and these patches are then fed into an encoder created based on the transformer to learn the deep feature representation in the image. The contextual features known by the transformer are then obtained using a decoder with patch extensions and fused with multiscale elements from the encoder *via* a jump connection to recover the spatial resolution of the feature images to further complete the translation of the target images. In our network, we also considered the global information of the picture to improve the performance of medical image translation. For images generated by the generator, we added deformable registration to our architecture to better train the generator in our network so that our model could yield better results in the image translation.

This study also has limitations, and further modifications to MMTrans are required for the practical implementation of medical imaging. Although computing with two-dimensional (2D) slices is significantly more efficient than the 3D counterparts, the information retained in the 3D data is indispensable for most medical imaging tasks. Therefore, future studies should further adapt MMTrans to 3D medical volumes.

### Conclusion

We present a novel Swin Transformer-based GAN for Multi-Modal Medical Image Translation, named MMTrans. First, the Swin Transformer-based generator with long-range dependency modeling ability is utilized for target image generation. Furthermore, a U-shaped registration network with Swin Transformer-based encoder is incorporated for better predicting deformable vector fields. Experimental results show that our MMTrans is superior to the existing MRI image-to-image translation methods and has great potential to be used in clinical practice.

## Data availability statement

Publicly available datasets were analyzed in this study. This data can be found here: http://www.med.upenn.edu/sbia/brats2018/data.html.

## Ethics statement

All subjects gave their informed consent for inclusion before they participated in the study with approval from the local institutional review board. The institutional review board (at Shanghai Ruijin Hospital) has approved the MRI scanning. Written informed consent was obtained from the individual(s) for the publication of any potentially identifiable images or data included in this article human studies are presented in this manuscript.

## Author contributions

Conceptualization: CW. Methodology: SY, JL, and CW. Software: SY. Validation: JL. Formal analysis: SY. Investigation: SY. Resources: CW. Data curation: SY. Writing—original draft preparation: SY. Writing—review and editing: SY, JL, and CW. Visualization: SY. Supervision: CW. Project administration: JL. Funding acquisition: JL and CW. Grammar proofreading: WC. All authors contributed to the article and approved the submitted version.

## Funding

This research was supported by the National Natural Science Foundation of China (Grant Numbers: 61902338, 62001120), and the Shanghai Sailing Program (Grant/Award Number: 20YF1402400).

## Conflict of interest

Author WC was employed by company Philips Healthcare.

The remaining authors declare that the research was conducted in the absence of any commercial or financial relationships that could be construed as a potential conflict of interest.

## Publisher’s note

All claims expressed in this article are solely those of the authors and do not necessarily represent those of their affiliated organizations, or those of the publisher, the editors and the reviewers. Any product that may be evaluated in this article, or claim that may be made by its manufacturer, is not guaranteed or endorsed by the publisher.
